# Two major-effect loci influence interspecific mating in females of the sibling species, *Drosophila simulans* and *D. sechellia*

**DOI:** 10.1093/g3journal/jkae279

**Published:** 2024-11-28

**Authors:** Kenneth Lu, Deniz Erezyilmaz

**Affiliations:** NYC Health and Hospitals/Lincoln, 234 East 149th Street, Bronx, NY 10451, USA; Centre for Neural Circuits and Behaviour, Department of Physiology, Anatomy and Genetics, University of Oxford, Mansfield Road, Oxford OX1-3SR, UK

**Keywords:** *sechellia*, assortative mating, pheromone, hybridization, behavioral isolation, prezygotic, invasive, endemism

## Abstract

Secondary contact between incompletely isolated species can produce a wide variety of outcomes. The vinegar flies *Drosophila simulans* and *D. sechellia* diverged on islands in the Indian Ocean and are currently separated by partial pre- and postzygotic barriers. The recent discovery of hybridization between *D. simulans* and *D. sechellia* in the wild presents an opportunity to monitor the prevalence of alleles that influence hybridization between these sibling species. We therefore sought to identify those loci in females that affect interspecific mating, and we adapted a two-choice assay to capture female mate choice and female attractiveness simultaneously. We used shotgun sequencing to genotype female progeny of reciprocal F1 backcrosses at high resolution and performed QTL analysis. We found 2 major-effect QTL in both backcrosses, one on either arm of the third chromosome that each account for 32–37% of the difference in phenotype between species. The QTL of both backcrosses overlap and may each be alternate alleles of the same locus. Genotypes at these 2 loci followed an assortative mating pattern with *D. simulans* males but not *D. sechellia* males, which mated most frequently with females that were hybrid at both loci. These data reveal how different allele combinations at 2 major loci may promote isolation and hybridization in the same species pair. Identification of these QTLs is an important step toward understanding how the genetic architecture of mate selection may shape the outcome of secondary contact.

## Teaser

For two species of *Drosophila*, interbreeding happens easily when the female is from the invasive cosmopolitan species *Drosophila simulans* and the male is from the island endemic species, *D. sechellia*. Lu and Erezyilmaz have used whole-genome mapping to identify regions of the genome that influence this species-specific mate choice. The authors find two regions on the third chromosome that have a profound effect upon the outcome of crosses between the two species and will play a role in ongoing hybridization in the wild.

## Introduction

Closely related species that have been evolving independently often retain the ability to interbreed, despite some degree of pre- or postzygotic isolation. When such divergent species come back into secondary contact, the outcome is difficult to predict. For cases where assortative mating signals are already established, reinforcement, which selects for premating isolation through selection against unfit hybrids, may complete the behavioral barrier between incipient species (Coyne and Orr 1989, 1997). In other cases, 2 species can blend through “de-speciation” (Turner 2002) or they may persist as long-term stable “hybrid swarms” (Barton and Hewitt 1989; Pfennig 2007; Servedio and Hermisson 2020). In some cases, 2 hybridizing species can also form an entirely new lineage, “homoploid hybrid speciation” (Mallet 2007; Lamichhaney *et al*. 2018). For instance, a Galapagos finch lineage became reproductively isolated after just 3 generations of hybridization (Lamichhaney *et al*. 2018). Intraspecific preferences for males with large beaks led females to favor an immigrant male of a related species. Therefore, mechanisms that govern mate choice within species can also facilitate hybridization between species.

Whether hybridization or species maintenance will follow secondary contact partly depends upon the sensory cues that related species use to recognize mates, and upon their genetic basis. One common feature among recently diverged species is the physical linkage between preference loci and loci that confer cues that are used as a basis for preference. For 3 separate *Heliconius* species pairs, the physical linkage between loci conferring preference for species-specific coloration with genes that determine species-specific coloration could contribute to recurrent species formation (Kronforst *et al*. 2006; Merrill *et al*. 2011; Rossi *et al*. 2020; Rosser *et al*. 2024). Indeed, the linkage between male courtship song and courtship song preference loci has also been found in *Laupala* crickets, a genus that has radiated new species more rapidly than any other invertebrate lineage that has been examined (Shaw and Lesnick 2009). In other cases, loci for assortative mating are physically linked to genes that confer adaptive differences, a feature that has been found in diverging populations of pea aphids (Hawthorne and Via 2001), stickleback fishes (Bay *et al*. 2017), and *Heliconius* butterflies (Merrill *et al*. 2011). The collective literature, therefore, indicates that the number and strength of loci that influence interspecific mating, as well as their location in the genome relative to other key loci, may contribute to the creation of new species or maintenance of existing ones.

The nascent species, *Drosophila simulans* and *D. sechellia*, are distinguished by host plant specialization and asymmetric behavioral isolation. *D. simulans* is a cosmopolitan generalist that probably first emerged in Madagascar and utilizes a wide variety of rotting fruit (Begun and Aquadro 1995; Andolfatto 2001; Dean and Ballard 2004; Kopp *et al*. 2006; Begun *et al*. 2007). *D. sechellia* is endemic to the Seychelles islands where it eats and breeds primarily on the fruit of *Morinda citrifolia*, which is toxic to all other Drosophila, including *D. simulans* (R'Kha *et al*. 1991). The 2 species will interbreed, but behavioral barriers affect males and females differently. *D. simulans* females and *D. sechellia* males readily mate in no-choice assays, but copulation rarely occurs in the reciprocal cross when *D. simulans* males are kept with *D. sechellia* females (Lachaise *et al*. 1986). Speciation between *D. simulans* and *D. sechellia* has progressed to partial postzygotic isolation since F1 male offspring are sterile although F1 females are fertile (Coyne *et al*. 1991).

Mate selection between this species pair occurs through a combination of both male and female choice, conferred through chemosensory and auditory cues, respectively. Contact pheromones are nonvolatile long-chain hydrocarbons that are secreted onto the cuticle. These cuticular hydrocarbons are produced by females of each species and are necessary for the barrier between *D. simulans* males and *D. sechellia* females (Coyne *et al*. 1994; Coyne and Oyama 1995; Billeter *et al*. 2009). An unsaturated 23 carbon with a double bond at C7, 7-Tricosene (7-T), is the predominant contact pheromone produced by *D. simulans* males and females, while *D. sechellia* females produce the 27-carbon compound, 7,11-heptacosadiene (7,11-HD) with a second double bond on C11 (Jallon and David 1987). 7,11-HD on females is detected by contact chemoreceptors on the male forelimb (Thistle *et al*. 2012). *D. simulans* males do not court *D. sechellia* females after contact unless the forelimb is removed (Shahandeh *et al*. 2018). Auditory cues also contribute to isolation between *D. simulans* and *D. sechellia*. Male fruit flies from the *D. simulans* complex “sing” species-specific courtship songs by vibrating their wings (Ritchie *et al*. 1999; Gleason and Ritchie 2004). *D. simulans* females mate more quickly when stimulated by the song of *D. simulans males* than *D. sechellia* males (Ritchie *et al*. 1999), and *D. sechellia* females are more likely to mate with *D. simulans* males if the males’ wings are removed. These data indicate that no song is better than producing the wrong song in inducing female receptivity (Tomaru *et al*. 2004).

Given the potential of this model of hybridizing species to reveal genetic contributions to interspecific mate selection, we have conducted this current study using whole genome shotgun genotyping to map those loci that prevent or facilitate interbreeding. We established a two-choice assay that uses copulation to assess isolation or hybridization between species. Both female preferences and those features that are used as a basis of preference by males will be selected for in our assay. We find that the genetic basis of between-species mating in females of these strains is localized to the third chromosome with 2 major-effect QTL, one on each arm of the third chromosome. Levels of interspecific mating are dependent upon different allele combinations at the 2 major loci. For instance, females with more *D. simulans* alleles at the 2 major QTL are more likely to mate with *D. simulans* males, but females bearing more *D. sechellia* alleles were not more likely to mate with *D. sechellia.* Instead, we found more affinity between females with hybrid genotypes at the 2 major-effect QTL and *D. sechellia* males. By understanding the effects these genotype combinations have upon interspecific mating of females in laboratory tests, we may next ask what effect they will have upon hybridizing populations in the wild.

## Materials and methods

### Drosophila strains

The *D. sechellia^13^* strain (originally obtained from the Tucson Stock Center #14021-0248.13) is derived from flies that were captured on Cousin Island in the Seychelles, an undeveloped nature reserve where *D. sechellia* was first discovered. *D. sechellia^D1A1C^* is an inbred strain that was produced by sib-mating single females of the *D. sechellia^13^* strain for 5 generations. The *D. simulans* Tsimbazaza strain (*D. simulans^Tsimba^*), which we obtained from David Stern (Janelia, HHMI), was originally isolated from Madagascar, where *D. simulans’* genetic variation is greatest and is probably where the species originated from (Begun and Aquadro 1995; Andolfatto 2001; Dean and Ballard 2004; Kopp *et al*. 2006; Begun *et al*. 2007). The *D. simulans^A2A2B^* line was produced by sib-mating single *D. simulans^Tsimba^* females for 5 generations.

### Two-choice assays

In these tests, females (either pure species, hybrids, or backcross progeny) were given the “choice” to mate with 1 of 2 species of males, *D. simulans* or *D. sechellia*. Virgin adult flies were sexed at eclosion and kept together in groups of 3 individuals of the same sex in standard Drosophila vials with media but without live yeast. We used females that were older than 3 days and as much as 28 days old because maximal mating of female *D. melanogaster* begins between 3 and 4 days after eclosion (Bilen *et al*. 2013). Male *D. melanogaster* peak in mating performance (multiple mating, short latencies, long durations of copulation, and high degree of fertility) between 1 and 4 weeks (Economos *et al*. 1979), so we used males that were between 7 and 28 days old in mate choice tests. It is not known if *D. simulans* and *D. sechellia* males age at the same rate, however, and we did not record the specific age of males. For the mating assays, live yeast paste was added to entirely empty fly food vials that did not contain any media. Flies were maintained at 25°C on a 12:12 light:dark cycle and the assays commenced within 1 h of subjective dawn. The assay began when 3 virgin females from selected strains or their hybrids were combined with 3 *D. simulans* males and 3 *D. sechellia* males. All 6 of the males in each test vial were aged the same number of days since eclosion. The assay was monitored for 4 h or until copulation, whichever came first. Once the first pair mated, the vial was moved to the freezer (−20°C), so that the pair were frozen *in copulo*. The species of copulating male was determined by the male genitalia, which is distinct for *D. simulans* and *D. sechellia* (Liu *et al*. 1996; Lu and Erezyilmaz 2022).

### Production of backcross populations

F1 females were first produced by crossing a *D. simulans^A2A2B^* female to a *D. sechellia^D1A1C^* male, because the reciprocal cross produces few offspring. F1 females were backcrossed to either *D. simulans^A2A2B^* males to produce *D. simulans* backcross progeny, or to *D. sechellia^D1A1C^* males to produce *D. sechellia* backcross progeny.

### Production of libraries for multiplexed shotgun genotyping

We used multiplexed shotgun genotyping (MSG) to infer the ancestry of individual female backcross progeny at hundreds of thousands of loci (Andolfatto *et al*. 2011; Cande *et al*. 2012). As described previously (Andolfatto *et al*. 2011), 10 ng of genomic DNA was extracted from individual flies in 96-well plates using the Puregene Tissue Kit (Qiagen, Venlo, the Netherlands), and labeled with one of 384 unique barcodes (Supplementary File 1). The 384 barcoded genomes were pooled for the final PCR and amplified together with modified Illumina flow cell primers (Andolfatto *et al*. 2011). Each Illumina library was comprised of 384 individual barcoded genomes (Supplementary File 2). Single-end 100 bp sequencing of the 3 MSG libraries, as well as the reference strains, *D. simulans^A2A2B^* and *D. sechellia^D1A1C^*, was performed on an Illumina Hi-Seq at the University of Oregon Genomics and Cell Characterization Facility (Eugene, Oregon).

### Genotyping of multiplexed libraries

The MSG package was installed on the cluster at NY Genome Center (New York, NY) to parse individual genomes and infer genome-wide ancestry for each fly. To create a more customized reference genome, we used the *D. simulans* w^501^genome assembly (Hu *et al*. 2013) as a scaffold and updated it with Illumina sequence reads from specific strains of the mapping lines, *D. sechellia^D1A1C^* and *D. simulans^A2A2B^* with the script, msgUpdateParental.pl, a part of the msg package (Andolfatto *et al*. 2011). The MSG software maps informative reads to 1 of 2 parental genomes and uses a hidden Markov model to compute a posterior probability that a genomic region is either homozygous for parent 1, homozygous for parent 2, or heterozygous (Andolfatto *et al*. 2011). The tab-separated values (.tsv) MSG-output files contain posterior probabilities, or “soft genotypes”, at hundreds of thousands of loci throughout the genome.

We then analyzed the 3 separate libraries comprised of 384 individuals with the MSG software and eliminated all individuals that were missing genotypes in stretches that include more than 15% of the genome. The individual files that were parsed according to barcode were combined into one large dataset and the entire set for each backcross was re-run together on the cluster at Janelia Farm (Ashburn, VA). The files of de-multiplexed individual reads and parental genomes are available on NCBI's Sequence Read Archive (SRA) under Bioproject PRJNA874916.

### Post-MSG processing

Changes in posterior probabilities between sequential markers indicate switches between genotypes (recombination breakpoints). We used the script pull_thin.py (Cande *et al*. 2012; Stern 2018) to thin our datasets to include only neighboring markers whose probabilities differed by at least 0.1, so that only markers that flank a recombination breakpoint in at least one individual are preserved. Data columns for markers that do not deviate from their neighbors for any individual were excluded from the output files. pull_thin.py also converts files to comma-separated value (.csv) output files that can be used by QTL analysis software. The MSG-output file of *D. simulans* backcross female progeny contained 782 individual genomes and was genotyped at 392,753 markers (∼1 marker/318 base pairs), then thinned to 3,461 markers (∼1 marker/36,106 base pairs). For the *D. sechellia* backcross, the file of 690 individual female genomes was genotyped at 392,427 markers (∼1 marker/318 base pairs), then similarly thinned to 2,890 markers (∼1 marker/43,240 base pairs). The comma-separated value files of genotype and phenotype are available at https://catalogue.ceh.ac.uk/documents/8dde3529-1cf7-4b0f-907d-a1631f38afd7.

### QTL mapping

A column for the choice phenotype of each female—which male she mated with in the two-choice tests—was added to the pull_thin.py csv output files. We used the R/qtl package (Broman *et al*. 2003) with R version 3.1.3 to analyze our backcross libraries. The function *scanone* was used to explore all possible single-QTL models, and *cim* was used for composite interval mapping (CIM) with the default values (window size = 10 cM and 3 markers). The binary model was used for each analysis. We used the Haley–Knott algorithm for interval mapping, CIM, and model building. 1,000 permutation replicates were used to assess the statistical significance of our log of the odds (LOD) scores (Doerge and Churchill 1996) for both the *scanone* and *cim* functions. Support intervals, the range of markers for which the likelihood ratio is roughly within a factor of 100, were inferred from a 2-LOD score drop from the peak LOD value (Lander and Botstein 1989). We used the *scantwo* function of r/qtl to perform a genome-wide scan of pairs of QTL on the full dataset, with 100 permutations to calculate the significance of our LOD scores. The effect of epistasis was shown by comparing the improvement of fit of the full model of over a single-QTL model (full-v-1) to the improvement of fit of the additive model over a single-QTL model (add-v-1). We explored the fit of multiple QTL models and pairs of interactions that were discovered by interval mapping, CIM and the scans for qtl pairs by using the functions, *fitqtl* and *sim.geno* with *step* = 1 cM (centiMorgan), *n.draws* = 128, and *err* = 0.001. We used a “drop-one” function to examine the contribution of individual loci and interactions. The best-fit model was found when additional loci reduced the variance of existing QTL without significantly increasing the LOD score of the model.

### No-choice tests and PCR-based genotyping of major QTL

No-choice tests, where females are combined with males of just one species, were performed to examine mate choice without competition between different species of males. Adult flies were isolated and kept in the same way as for the two-choice tests, but in this case males from just one species were combined with 3 backcross females. For these tests, we ended the trial after 2 h, rather than 4 since we learned that nearly all mating occurred within the first 2 h. The *D. sechellia^D1A1C^* strain was lost after the QTL maps were produced, so the backcross progeny for the no-choice tests were produced with the strain *D. sechellia^13^* and the inbred line *D. simulans^A2A2B^*. We used the primer pairs *desatF_for/desatF_rev* (melting temperature, Tm = 60°C) and *eloF_for/eloF_rev* (Tm = 62°C) with MyTaq Red (Bioline, Meridian Bioscience) to distinguish between *D. simulans* and *D. sechellia* alleles at the major QTL on 3L and 3R, respectively. The primer sequences *eloF* 5′ CAACATATTCCAGATCCTTTACAA3′ e*loF*-R 5′ ATCCTTATATTTGTGATCCATCG3′, which amplify a species-specific sequence length polymorphism at 3R: ∼15,390,000 base pairs (3R: ∼15.39 Mega base pairs, Mbp). The primers *desatF*-F 5′CCTGAACACTTTGGCCTTCC3′ and *desatF*-R 5′ATTTGCTTGCCCTTCTCCAC3′ (Swartzlander 2016), amplify a species-specific sequence length polymorphism at 3L: ∼10.76 Mbp (Supplementary File 8). We used Prism software to assess the significance of associations in 2 × 2 Contingency Tables using Fisher's Exact Test genotype frequencies between mating phenotypes. The ratio of *i/e; i/e* heterozygote females that mated with *D. sechellia* to those that did not, was compared to the ratio of *e/e; e/e* homozygote females that mated with *D. sechellia* to those that did not (Fig. 5d).

## Results

We devised a high throughput method to screen for alleles that contribute to hybridization between *D. simulans* and *D. sechellia*. We used a two-choice assay, a standard test of behavioral isolation (Boake *et al*. 2003; Yukilevich and True 2008; Bay *et al*. 2017), instead of a no-choice assay because interspecific mating occurs more readily when flies are not given the option to mate with their own species (Coyne *et al*. 2005). This approach has the advantage that each “choice” represents a positive unambiguous score of behavioral isolation; however, the two-choice design cannot distinguish female preference traits from the traits that confer female attractiveness to males. Copulation was too infrequent when females were tested one-by-one, so 3 females were combined with 3 *D. simulans* males and 3 *D. sechellia* males for each mating trial, and these were observed for 4 h. Fruit flies reproduce on ripe fruit that is fermented by yeast (Becher *et al*. 2012) and we included live yeast paste in the test vial because it is a natural cue for reproduction (Gorter *et al*. 2016).

**Fig. 1.**
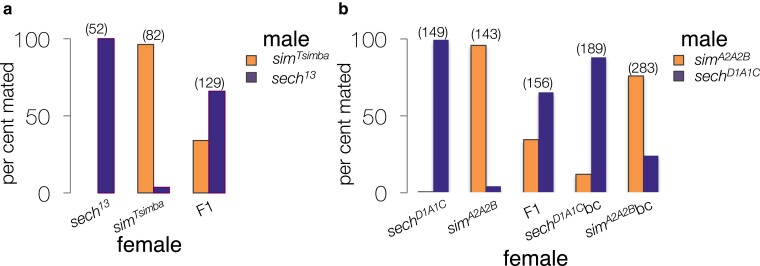
Outcomes of two-choice tests of assortative mating. Each test vial contained: (1) 3 test females (either pure species, inbred lines, F1 females, or F1 backcross progeny), (2) 3 *D. sechellia* males, and (3) 3 *D. simulans* males. The number of copulating pairs is given in parentheses above each pair of bars. a) The percent of mate choice tests that resulted in female mating with males of *D. simulans* from the Tsimbazaza strain (*sim^Tsimba^*, tangerine-colored bars, the bars to the left in each pair) or the *D. sechellia^13^* strain (*sech^13^*, plum-colored bars, the bars to the right of each pair) is given on the *y*-axis. The *x*-axis indicates the females that were used in the two-choice tests. F1 = female progeny from a *D. simulans^Tsimba^* mother and a *D. sechellia^13^* father. b) The lines *D. simulans^A2A2B^* (*sim^A2A2B^*) and *D. sechellia^D1A1C^* (*sech^D1A1C^*) were derived from *D. simulans^Tsimba^* to *D. sechellia^13^*, respectively, by brother–sister mating for 5 generations. The percent of mate choice tests that resulted in female mating with males of *D. simulans^A2A2B^* (tangerine-colored bars) or the *D. sechellia^D1A1C^* strain (plum-colored bars) is given on the *y*-axis. The *x*-axis indicates which females were used in the two-choice tests. F1 hybrid females (*D. simulans^A2A2B^*/*D. sechellia^D1A1C^*) were backcrossed to *D. simulans^A2A2B^* males or *D. sechellia^D1A1C^* to generate the F1 backcross progeny females that were used in the two-choice tests (*sech^D1A1C^*bc or *sim^A2A2B^*bc).

We examined the frequency of interspecific mating in females of the pure species, inbred lines, hybrids, and backcross progeny (Fig. 1a). *D. simulans* is a cosmopolitan species and behavior is known to differ between New-World *D. simulans* and *D. simulans* strains taken from Madagascar (Erezyilmaz and Stern 2013), which is where *D. simulans* probably evolved (Begun and Aquadro 1995; Andolfatto 2001; Dean and Ballard 2004; Kopp *et al*. 2006; Begun *et al*. 2007). We used a strain of *D. simulans* from Madagascar, *D. simulans^Tsimba^*. Copulation between a *D. sechellia^13^* female and a *D. simulans^Tsimba^* male was very rare in the two-choice test, but *D. sechellia* males and *D. simulans* females were first to copulate in approximately 5% of the trials. We found that F1 hybrid female progeny, from a *D. simulans^Tsimba^* mother and a *D. sechellia^13^* father, mate more frequently with *D. sechellia^13^* males by nearly a 2:1 ratio indicating partial dominance for this trait (Fig. 1a). These results resemble what has been previously reported with other strains (Lachaise *et al*. 1986; Cobb and Jallon 1990; Coyne 1992). Within-species genetic variation could contribute to behavioral variation and complicate our mapping so we reduced it by sib-mating the *D. simulans^Tsimba^* strain to itself for 5 generations and the *D. sechellia^13^* strain to itself for 5 generations. We then confirmed that the same phenotypes hold in our mapping strains, *D. simulans^A2A2B^* (derived from *D. simulans^Tsimba^*) and *D. sechellia^D1A1C^* (derived from *D. sechellia^13^*). While 0 of 149 *D. sechellia^D1A1C^* females mated with *D. simulans^A2A2B^* males, some *D. simulans^A2A2B^* females mated with *D. sechellia^D1A1C^* males, reflecting the pattern that was observed in the pure species (Fig. 1b). We backcrossed F1 females to produce 189 *D. sechellia* backcross progeny and separately, 283 female progeny of the *D. simulans* backcross. More females mated with males that were the same species as their father (Fig. 1b).

### QTL mapping of interspecific mating in females of the *D. simulans* backcross

**Fig. 2.**
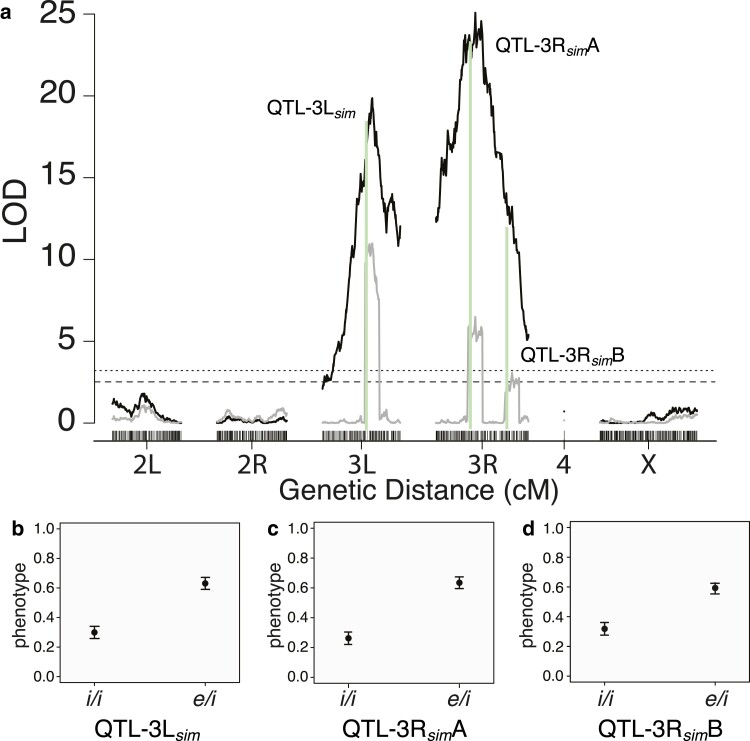
QTL analysis of the *D. simulans* backcross. a) Results of interval mapping (black lines) and CIM (gray lines) of interspecific mating of female progeny. Chromosome markers are given on the *x*-axis over genetic distance with LOD on the *y*-axis. The horizontal dashed lines indicate the *P* = 0.05 genome-wide significance threshold, and the dotted line shows the *P =* 0.01 significance threshold, for both the interval mapping and CIM. Green vertical lines indicate the location of QTL peaks and their markers. The peak value on 3L, QTL-3L_*sim*_, is at 3L: ∼11.31 Mbp, and a 2-LOD support interval, where the LOD score is within 2-LOD of the peak maxima, spans from 3L: ∼10.66 to 11.92 Mbp. The highest value QTL on 3R, QTL-3R_*sim*_A, is at 3R: ∼14.75 Mbp, and the 2-LOD support interval spans from 3R: ∼14.14 to 15.06 Mbp. QTL-3R_*sim*_B, found in the CIM, was detected at 3R: ∼22.20 Mbp. b–d) Effect plots showing the phenotypic effect of substituting *D. sechellia* allele “*e*” for a *D. simulans* allele “*i*”. The “phenotype” on the *y*-axis is the propensity to mate with *D. sechellia;* 0 = mating with a *D. simulans* male and 1 = mating with a *D. sechellia* male at QTL-3L_*sim*_ b), QTL-3R_*sim*_A c), and QTL-3R_*sim*_B d). The error bars show the standard error.

A second cohort of female progeny of the *D. simulans* backcross was tested in the two-choice assay and genotyped using MSG (Andolfatto *et al*. 2011). We collected 415 recombinant backcross females that mated with *D. simulans* males and 367 recombinant backcross females that had mated with *D. sechellia* males. Individual females were genotyped using the MSG protocol, and the choice of male was treated as a binary trait in QTL analysis. QTL mapping reveals 2 significant QTL peaks: one centered at 3L: ∼11.31 Mbp (QTL-3L_*sim*_), LOD = 19.86, with a 2-LOD drop confidence interval between 3L: ∼10.66 and 11.92 (Fig. 2a; Supplementary File 3). Another larger peak (LOD = 25.08) is centered over a marker near 3R: ∼14.75 Mbp (QTL-3R_*sim*_A), within a 2-LOD drop confidence interval 3R: ∼14.14–15.06 (Fig. 2a; Supplementary File 4). The entire third chromosome was significant, so we next performed CIM (gray line, Fig. 2a). CIM combines multiple regression with interval mapping to control for multiple neighboring loci to reduce residual variation (Zeng 1994), although in some cases this method can lead to reduced detection power and false confidence in QTL location (Broman and Sen 2009). The 2 major peaks that were discovered in our interval mapping approach co-localized with the 2 major loci discovered with CIM, suggesting that the estimated location of the 2 major QTL is not obscured by the effects of neighboring loci. CIM indicates an additional significant locus is found near the distal end of the chromosome, 3R: ∼22.15 Mbp (QTL-3R_*sim*_B; LOD = 3.07), which is within the 2-LOD drop confidence interval, 3R: ∼21.29–22.99 Mbp (Supplementary File 5). The effect of each locus is considerable; substitution of a *D. sechellia* allele at the locus on 3L results in 32% increase in the likelihood of hybrids mating with a *D. sechellia* male (Fig. 2b) and substitution of a *D. sechellia* allele at the major locus at 3R: ∼14.75 Mbp increases the likelihood of hybrid females mating with *D. sechellia* males by 37% (Fig. 2c). At 3R: ∼22.15 Mbp, substitution of a *D. sechellia* allele has an effect of 22.3% (Fig. 2d). When we compared the relative mating rates of females of different genotypes at QTL-3L_*sim*_ and QTL-3R_*sim*_A between *D. simulans* and *D. sechellia* males (Fig. 4a), we saw that females mated assortatively. That is, females with more *D. sechellia* alleles at QTL-3L_*sim*_ and QTL-3R_*sim*_A (represented by “*e*”) mated with *D. sechellia* males at a greater rate, while females with more *D. simulans* alleles (represented by “*i*”) mated with *D. simulans* males at a greater rate (Fig. 4a).

We also considered all possible two-QTL models in the *D. simulans* backcross, with and without epistasis. Genome-wide scans confirm those QTL discovered in the single-QTL scans, QTL-3L_*sim*_, QTL-3R_*sim*_A, and QTL-3R_*sim*_B (Supplementary Fig. 1). The two-QTL scans also detected epistasis between 2 loci on the right arm of the third chromosome: 3R: ∼16.23 × 3R: ∼7.87 Mbp, LOD_*full*_ = 30.1, LOD_*full-v-1*_ = 6.94, and LOD_*int*_ = 2.80 (Supplementary Fig. 1a), and another additive pair of loci, 3R: ∼16.23 × 3R: ∼5.54 Mbp (LOD_*add*_ = 27.3, LOD_*add-v-1*_ = 4.14). However, when we examined the fit of combinations of loci together in multiple QTL models (Supplementary Table 1), the best-fit model includes just the 3 major-effect loci that were identified in our interval mapping, 3L: ∼11.31 Mbp (QTL-3L_*sim*_), 3R: ∼14.76 Mbp (QTL-3R_*sim*_A), and 3R: ∼22.15 Mbp (QTL-3R_*sim*_B; Table 1), with a modest interaction between QTL-3L_*sim*_ and QTL-3R_*sim*_B (Fig. 2a; Table 1; Supplementary Table 1).

**Table 1.** The QTL favored models of the *D. simulans* backcross (*bc-sim*) and the *D. sechellia* backcross (*bc-sech*).

Cross	# QTL	% variance (model)	LOD of model	QTL location (Mbp)	LOD drop one*^a^*	% variance (qtl)
*bc-sim*	3 + interaction	20.04	38.01	3L: ∼11.31	12.06	6.55
				3R: ∼14.76	6.11	3.28
				3R: ∼22.15	4.61	2.51
				3L: ∼11.31 × 3R: ∼22.15	1.59	1.05
*bc-sech*	4 + interaction	20.38	34.14	3L: ∼10.35	13.61	7.57
				3R: ∼16.63	1.66	0.89
				3R: ∼11.49	2.95	1.58
				3R: ∼26.41	4.42	2.39
				3R: ∼11.49 × 3R: ∼26.41	1.55	0.83

^*a*^Log-likelihood ratios comparing the full model to a model with the specified QTL removed.

### Alleles that influence interspecific mating in females of the *D. sechellia* backcross

**Fig. 3.**
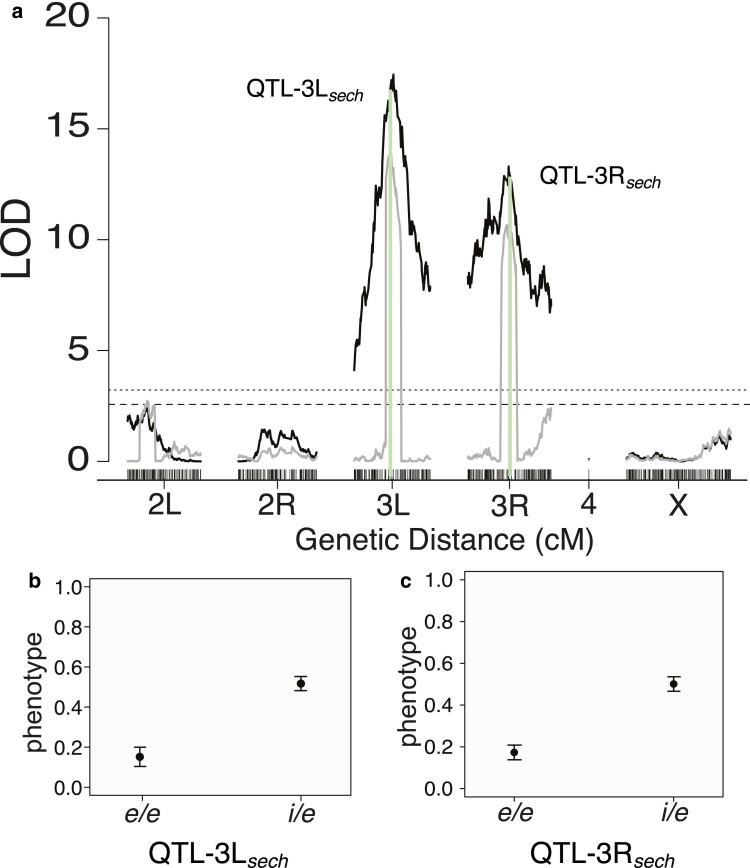
QTL analysis of the *D. sechellia* backcross. a) Results of interval mapping (black lines) and CIM (gray lines) of interspecific mating of female progeny. Chromosome markers are given on the *x*-axis over genetic distance with LOD on the *y*-axis. The horizontal dashed lines indicate the *P* = 0.05 genome-wide significance threshold, and the dotted line shows the *P =* 0.01 genome-wide significance threshold for both the interval mapping and CIM. Green vertical lines indicate the location of QTL peaks and their markers. The peak LOD score on 3L is at 3L: ∼10.48 Mbp, QTL-3L_*sech*_ and the 2-LOD support interval spans from 3L: ∼8.53 to 11.90 Mbp. For 3R, the peak lies at 3R: ∼16.65 and spans from 3R: ∼14.55 to 17.43 Mbp (QTL-3R_*sech*_). b, c) Effect plots showing the phenotypic effect of substituting a *D. simulans* allele “*i*” for a *D. sechellia* allele “*e*” where phenotype is 0 = mating with a *D. sechellia* male and 1 = mating with a *D. simulans* male at QTL-3L_*sech*_ b) and QTL-3R_*sech*_ c). The standard error is shown in the error bars.

We repeated the two-choice assay for a new cohort of female progeny of the *D. sechellia* backcross. DNA of individual genomes were extracted in 96-well plates, sequenced, and processed using the MSG pipeline as we had done for the *D. simulans* backcross. We performed interval mapping of 287 female progeny of the *D. sechellia* backcross that had mated with *D. simulans* and 403 that had mated with *D. sechellia* males (Fig. 3a, dark lines). We found that, like the *D. simulans* backcross, the effect of female interspecific mating is largely restricted to chromosome 3, with a strongly significant peak on each arm of the third chromosome. One highly significant locus on 3L at ∼10.75 Mbp (QTL-3L_*sech*_; LOD = 17.44) with a 2-LOD drop CI between 3L: ∼8.53 and 11.90 Mbp (Supplementary File 6), and another on the right arm of the chromosome, 3R: ∼16.63 Mbp, (QTL-3R_*sech*_; LOD = 11.68; Fig. 3a) with a 2-LOD drop between 3R: ∼14.55 and 17.43 Mbp (Supplementary File 7). CIM (Fig. 3a, gray lines) supports the same 2 major QTL peaks on 3L and 3R but does not indicate additional significant QTL. As with the QTL from the *D. simulans* backcross, the 2 major loci were of considerable effect. The substitution of a *D. simulans* allele at QTL-3L_*sech*_ had a 36.5% effect upon the likelihood of mating with *D. simulans* (Fig. 3b), and the effects of substitution at QTL-3R_*sech*_ had an effect of 32.8% (Fig. 3c). The similarity between the QTL map for mate choice in the *D. simulans* backcross and the *D. sechellia* backcross could suggest that alleles of the same genes influence mate choice in both backcross populations (Supplementary Fig. 2, a and b), although there is additional complexity on the distal end of 3R, perhaps due to the presence of an additional locus at the distal end that is only resolved into QTL in the *D. simulans* backcross (Fig. 2a; Supplementary Fig. 2).

**Fig. 4.**
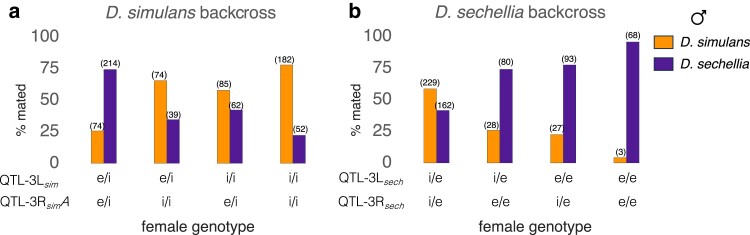
The percentage of females of each genotype at the 2 major QTL that mate *D. simulans* males (tangerine bars) or *D. sechellia* males (plum-colored bars) in the a) *D. simulans* backcross or b) *D. sechellia* backcross mapping population. The number of females of each genotype category is given above each column. The genotype at 3L: 11,311,968 was used as a marker of QTL-3L_*sim*_ and the genotype at 3R: 14,758,494 was taken for QTL-3R_*sim*_A, which are at the peak values from single-QTL interval mapping. Similarly, we used the genotype at 3L: 10,480,434 as the marker for QTL-3L_*sec*_, and the genotype at 3R: 16,634,292 as a marker for QTL-3R_*sec*_. *e* = *D. sechellia* allele and *i* = *D. simulans* allele of each QTL.

We next compared the rate of mating to males of either species for females of each genotype class of the *D. sechellia* backcross mapping population (Fig. 4b). We saw that fewer *e/e; e/e* homozygote females mated with either species’ male than was expected; of 690 females that mated in the two-choice tests of *D. sechellia* backcross females, only 71 mated. Of the 71 *e/e; e/e* females that did mate, only 3 had mated with a *D. simulans* male. We also saw that assortative mating was less strong between *D. sechellia* males and females that were homozygous for *D. sechellia* alleles at QTL-3L_*sec*_ and QTL-3R_*sec*_, since more than twice as many (*i/e; i/e*) females mated with *D. sechellia* males (*e/e; e/e*, Fig. 4b). Only females that mated were genotyped, however, so we cannot exclude the formal possibility that far fewer (*e/e; e/e*) females were available for mating in the assay.

Scans for pairs of QTL in the *D. sechellia* backcross showed that the highest LOD scores are also found on the third chromosome but placed QTL-3L_*sech*_ at a slightly different location in the interaction: 3L: ∼10.35 × 3R: ∼16.63 Mbp (LOD_*full*_ = 29.3, LOD_*int*_ = 0.329, LOD_*add*_ = 28.93). The scan for interacting pairs also suggested an interaction between 2 loci on the right arm of the third chromosome, 3R: ∼11.49 × 3R: ∼26.41 Mbp (LOD_*full*_ =19.2, LOD_*int*_ = 0.99, LOD_*add*_ = 18.74). Allowing epistasis made little difference in LOD scores overall (Supplementary Fig. 1b).

Because each analysis to this point implicates multiple regions on the third chromosome, we next considered the fit of candidate loci in multiple QTL models (Supplementary Table 2). The best-fit model (LOD = 34.14) includes the 2 major QTL on either arm 3L: ∼10.35 Mbp (QTL-3L_*sec*_) and 3R: ∼16.63 Mbp (QTL-3R_*sec*_), as well as the 2 smaller loci at 3R: ∼11.49 and 3R: ∼26.41 Mbp, and a modest interaction between them (Table 1; Supplementary Table 2).

### Tests of major QTL effects upon hybridization of females in no-choice mating tests

The effects of each QTL on mating rate, competition, or differences in the propensity to mate could be masked in a two-choice test. For instance, copulation with males of one species in a two-choice test cannot distinguish whether a female is more attractive to males of one species, or whether it is disfavored by males of the other species. We therefore examined allele frequency of the major QTLs on 3L and 3R in samples of 3 backcross females that were paired with 3 males from only one species. This would discern whether a female genotype confers a positive or negative effect upon mating with males of either species. In contrast to the interspecific mating tests for our mapping population when we only genotyped those females that mated, we also genotyped those females that failed to mate in this experiment. In the QTL analysis of both backcrosses, the gene *desatF* is located within the composite interval of QTL-3L, and the gene *eloF* is located within the composite interval of QTL-3R (Supplementary Fig. 2). We used polymorphisms in these genes as imperfect proxies for the *D. simulans* and *D. sechellia* alleles of QTL-3L and QTL-3R by modifying an established PCR assay (Swartzlander 2016).

**Fig. 5.**
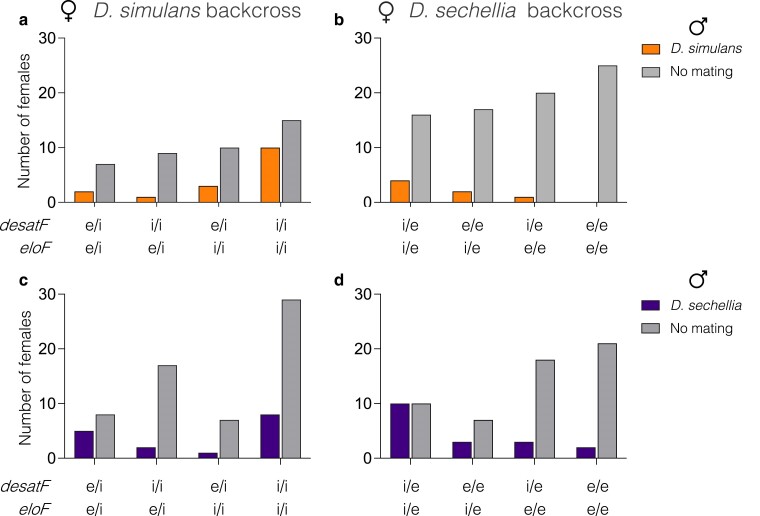
Frequency (*y*-axis) of mating with males in a no-choice assay. Three female backcross progeny of random genotypes were combined with 3 males (either *D. simulans* or *D. sechellia*). All females were genotyped using species-specific polymorphisms at *desatF* (near QTL-3L) and *eloF* (near the major QTL on 3R), and the genotypes are given on the *x*-axis where “*i*” represents the *D. simulans* allele and “*e*” represents the *D. sechellia* allele. The number of females with each genotype that mated with *D. simulans* males is given in the tangerine-colored bars or *D. sechellia* males (plum color). The frequency of females with a given genotype that did not mate in the trials is shaded gray. a) Frequency of *D. simulans* backcross genotypes mating with *D. simulans males*, b) frequency of *D. sechellia* backcross genotypes mating with *D. simulans* males, c) frequency of *D. simulans* backcross genotypes mating with *D. sechellia* males, and d) frequency of *D. sechellia* backcross genotypes mating with *D. sechellia* males.

Crosses between female progeny of the *D. sechellia* backcross with males of either species are characterized by low mating rates among all genotype classes, and very strong repellence between some female genotypes and *D. simulans* males. Females that were homozygous for the *D. sechellia* alleles at both loci were the least likely to mate with either *D. simulans* or *D. sechellia*. No females that were *e/e; e/e* at *desatF; eloF* (QTL-3L_*sech*_; QTL-3R_*sech*_) mated with *D. simulans* males (Fig. 5b), and strikingly, only about 9% of *e/e; e/e* females mated with *D. sechellia* males (Fig. 5d). Moreover, in tests of females from the *D. sechellia* backcross with *D. sechellia* males, females that were heterozygous at *desatF* and *eloF* (*i/e; i/e*) mated at the greatest rate (Fig. 5d), even compared to *e/e; e/e* homozygote females (*P* = 0.005, two-tailed FET). Together, these results reveal that copulation with any male is less frequent for females that are homozygous for the *D. sechellia* alleles at *desatF* and *eloF*. For such females, a lack of mating apparently plays a larger role than competition for or between males.

The mating pattern of females from the *D. simulans* backcross was more assortative. Females of all QTL-3L_*sim*_ and QTL-3R_*sim*_A genotypes would mate with males of either species, but at different rates (Fig. 5, a and c). In tests with *D. simulans* males, females that were homozygous for *D. simulans* alleles at both QTL-3L_*sim*_ and QTL-3L_*sim*_A (*i/i; i/i*) were most successful. In tests with *D. sechellia* males, females that were heterozygous (*e/i; e/i*) mated at a greater rate than females that were homozygous for the *D. simulans* alleles at either or both QTL.

## Discussion

The sibling species *D. simulans* and *D. sechellia* have been an important model of speciation, and with the report of ongoing hybridization in the Seychelles (Matute and Ayroles 2014), they provide an opportunity to study species maintenance. Previous studies have examined the genetic basis of reproductive isolation between *D. simulans* and *D. sechellia*. Coyne (1992) used insemination to map isolation of females to the second and third chromosomes, and Chu *et al*. (2013) mapped the production of offspring to the X, second, and third chromosomes. We restricted our analysis to the behavioral components of isolation, with female choice and female attractiveness in the two-choice assay. We used a *D. sechellia* strain derived from Cousin Island, and a *D. simulans* strain derived from Madagascar, where *D. simulans* is endemic. We found that much of the genetic variation in interspecific mating in these females is localized to the third chromosome with 2 major loci, one on each arm. Each of the 2 major-effect QTL for interspecific mating is found in both backcross directions and individually each account for 32–37% of the difference in phenotype between species, which is of considerable magnitude for a behavioral trait (York 2018).

### Genetic contributions to the major QTL

The strength of the 2 major QTLs in the *D. simulans* backcross occurs through differences in the relative mating rates between female genotypes and males of either species. Hybrid females that are homozygous for the *D. simulans* alleles at QTL-3L_*sim*_ (*desatF*) and QTL-3R_*sim*_A (*eloF*) were more likely to copulate with *D. simulans* males than females bearing a *D. sechellia* allele at either locus or both loci (Figs. 4a and 5, a and b). By contrast, the outcome of two-choice tests with females of the *D. sechellia* backcross was shaped by whether the females of a given genotype tend to mate with either species’ male at all. We only know the QTL-3L_*sech*_; QTL-3R_*sech*_ genotypes of females that mated in our mapping population, but just 71 of 690 copulations were with *e/e; e/e* females, compared to 391 copulations with *i/e; i/e* females. Of those 71 copulations with *e/e; e/e* females, only 3 were with *D. simulans* males (Fig. 4b). In the no-choice tests where we knew the genotypes of all females, those that were homozygous for the *D. sechellia* allele at both *desatF* (near QTL-3L_*sech*_) and *eloF* (near QTL-3R_*sech*_) had the lowest mating frequency with either male (Fig. 5, b and d). Such *e/e; e/e* females never copulated with *D. simulans* males in the 25 no-choice tests, and the recombinant females (*i/e; e/e* or *e/e; i/e)* also mated at a very low rate when paired with *D. simulans* males (Fig. 5b). So, although females bearing homozygous *D. sechellia* alleles at the 2 major loci mate with *D. sechellia* males at a low rate, copulation between those females and *D. simulans* males is far less frequent, and this difference can explain the strength of QTL-3L_*sech*_ and QTL-3R_*sech*_.

Another feature of the mating pattern between these species is that F1 hybrid females were more likely to mate with *D. sechellia* than with *D. simulans* males by a 2:1 ratio in the two-choice tests, (Fig. 1, a and b). The results of our choice tests in the backcross progeny indicate that the affinity between *D. sechellia* males and hybrid females is at least partly localized to QTL-3L_*sec*_ (*desatF*) and QTL-3R_*sec*_ (*eloF*). Specifically, *i/e; i/e* heterozygote females produced by the *D. sechellia* backcross were more likely to mate with *D. sechellia* males and in fact did so at a greater rate than with *e/e; e/e* homozygote females (Figs. 4b and 5d). Preference for hybrids has been observed in multiple taxa and hybridization contributes genetic diversity (Lesna and Sabelis 1999; Veen *et al*. 2001; Grant and Grant 2002; Pfennig 2007; Lamichhaney *et al*. 2018). For wild populations found in the hybrid zones of the Seychelles, all males that were identified as hybrid bore the genotype of a *D. simulans* mitochondria and a *D. sechellia* Y chromosome, indicating that (1) the initial hybridization was between a *D. simulans* female and a *D. sechellia* male and (2) the same affinity between hybrid females and *D. sechellia* males is also a factor in wild populations (Matute and Ayroles 2014). Our results show that such affinity between the hybrid females and *D. sechellia* males should persist among wild populations beyond the initial F1- or first backcross stages as long as those females are heterozygous for the *D. simulans/D. sechellia* alleles at the major QTL on 3L and 3R.

### Parallels with the QTL map for 7,11-HD production

Our screen was designed to capture the loci that contribute signals for male preference and the loci that confer mate choice in females. However, the composite interval QTL map produced by our QTL analysis of interspecific mating in females bears a strong resemblance to a QTL map of variation in 7,11-HD production between *D. simulans* and *D. sechellia* females (Coyne *et al*. 1994; Gleason *et al*. 2005, 2009). For Gleason *et al*. (2005, 2009), 2 major QTLs contribute to variation in 7,11-HD levels in the *D. simulans* backcross: one between markers near 3L: 10.85 Mbp and 3L: 11.88 Mbp, and a second between markers near 3R: 13.89 Mbp and 3R: 15.42 Mbp. These 7,11-HD QTL are near the major QTL that we have found that affect interspecific hybridization in females (3L: ∼11.31 Mbp and 3R: ∼14.76 Mbp), as well as with 2 enzyme-encoding genes that are required for conversion of 7-T to 7,11-HD, *eloF* (Chertemps *et al*. 2007; Combs *et al*. 2018), and *desatF* (Chertemps *et al*. 2006; Shirangi *et al*. 2009). Genes for both enzymes are expressed in *D. sechellia* females but not in males, or in either sex of *D. simulans* (Chertemps *et al*. 2006, 2007; Shirangi *et al*. 2009).

Is the barrier between *D. simulans* males and *D. sechellia* females due entirely to male *D. simulans’* aversion to 7,11-HD produced by *D. sechellia* females? We do not believe so. First, crosses between *D. simulans* and *D. melanogaster* are more successful when male *D. simulans* is paired with female *D. melanogaster*, which also produce 7,11-HD. But the reciprocal cross, with *D. simulans* females and *D. melanogaster* males, is rarely successful (Carracedo *et al*. 1998). If *D. simulans* males are entirely thwarted by 7,11-HD, they would be similarly thwarted in crosses with *D. melanogaster* females. Secondly, many studies have shown that female Drosophila perceive male courtship song, and some have found that species-specific songs matter (Ritchie *et al*. 1999; Tomaru *et al*. 2004). It is therefore likely that additional signals contribute to the likelihood of hybridization.

### A complex genetic architecture on the third chromosome

Although peaks of the 2 major loci, QTL-3L and QTL-3R, are the 2 largest genetic factors to contribute to the interspecific mating in females, it is clear from the QTL map that other loci on the third chromosome contribute as well. Loci that confer aspects of isolation between species are often found clustered together in the genome (Byers *et al*. 2021), and the breadth of the major peaks could mean that linked loci contribute to the outcome of females’ interspecific mating (Supplementary Fig. 1a). Also, there is additional complexity on the distal end of 3R in the maps of both backcrosses (Figs. 2a and 3a; Supplementary Fig. 1). Several genes encoding desaturases and elongases that are related to *desatF* and *eloF* are found on the distal half of 3R, and many of these are differentially expressed between the 2 species (Combs *et al*. 2018). In the case that differences in 7,11-HD production underlies the QTL of our map, genes encoding similar enzymes could also contribute to overall differences in cuticular hydrocarbon pheromones, and it would account for the particularly high LOD scores found on the right arm of chromosome 3.

One candidate locus conferring female preference has also been mapped to the third chromosome in *D. simulans*. The *fruitless* (*fru*) gene is a “master-regulator” of male sexual behavior in *D. melanogaster*. Deletions in the *fru* gene that unmask the *D. simulans* allele in *D. simulans/D. melanogaster* F1 hybrid females reveal that this locus confers female preference for *D. simulans* males and rejection of *D. melanogaster* males (Laturney and Moehring 2012; Chowdhury *et al*. 2020). Our QTL maps do show a minor rise in LOD score around the *fru* locus (∼7 Mbp, Supplementary Fig. 2), and our two-QTL scan of the *D. simulans* backcross found some evidence for a QTL near *fru* (Supplementary Fig. 1a). However, this region is not significant in either CIM or in multiple QTL models (Supplementary Fig. 1; Table 1). The discrepancy between our study and Chowdhury *et al* (2020) could be due to differences in our assays, since they used transmission of sperm to test for isolation between species. Or perhaps for *D. simulans* females, *fru* imparts rejection of *D. melanogaster* males through a mechanism that is distinct from rejection of *D. sechellia* males. It is also possible that the QTL results that we have discovered here may be specific to our mapping strains only, since Chowdhury *et al* (2020) used 3 strains of *D. simulans* that are different from the one used in this study.

### Linkage with other traits affecting interspecific mate selection

The physical linkage between loci conferring a preference and loci conferring a preference cue may be a common feature separating diverged species (Kronforst *et al*. 2006; Pryke 2009; Shaw and Lesnick 2009; Merrill *et al*. 2011; Rossi *et al*. 2020). Without linkage, the 2 traits are expected to dissociate through recombination. We found some linkage between the loci that confer species-specific male courtship behavior and those loci that influence hybridization between *D. simulans* and *D. sechellia* in females. Work over decades has localized variation in aspects of mate choice by males for this species pair to the right arm of chromosome 3, including species-specific male courtship song (Gleason and Ritchie 2004), male mating success, courtship latency (Cantor and Civetta 2003), and courtship vigor, which is the difference in male courtship index that is directed toward females of either species (Shahandeh and Turner 2020). Fine mapping of courtship vigor has identified a cluster of 3 loci at 3R: ∼9.2–10.1 Mbp, 3R: ∼11–13.9 Mbp, and 3R: ∼20.0 Mbp that together account for approximately 44% of the variation in this trait. These courtship vigor QTL of males are found in, and among the loci that we have identified on 3R, including QTL-3R_*sim*_A (3R: ∼14.76 Mbp) and QTL-3R_*sech*_ (3R: ∼16.63 Mbp) and the 2 regions discovered in the two-QTL scan of the *D. sechellia* backcross, 3R: ∼11.49 × 3R: ∼26.41 Mbp. Therefore, the major-effect loci underlying between-species mating of males are interspersed among the major-effect loci that influence interspecific mating of females in a ∼12 Mbp stretch on 3R. It is not clear from this study, however, if the proximity between loci is extensive enough to affect species integrity as predicted by theory (Felsenstein 1981).

Behavioral isolation between *D. simulans* and *D. sechellia* may be complicated if 2 different types of cues with 2 corresponding preference behaviors are used to identify mates: an auditory cue produced by males that is perceived in females, and a chemosensory cue produced by females that is perceived by males. Two distinct mate recognition systems might reinforce species’ barriers, or the 2 systems could also direct other outcomes. For the Galapagos finch lineage that became reproductively isolated after just 3 generations, female preference for large beaks drove hybridization with an immigrant species (Price 1984), yet mate song preference learned from the immigrant father drove isolation within the offspring (Lamichhaney *et al*. 2018). If 2 mate selection mechanisms influence *D. simulans–D. sechellia* hybridization, the genetic architectures of species-specific courtship song in males, courtship song preferences of females, contact pheromone production of females, and contact pheromone preferences of males could each contribute to the outcome of hybridization in ways that are similarly difficult to predict.

In addition to differences in preferences and preference cues encoded in the genome, loci that confer adaptation to host fruit may indirectly affect mate selection between *D. simulans* and *D. sechellia*. Courtship and mating in vinegar flies occurs on ripe and rotting fruit. Females lay eggs in host fruit, which hatched larvae feed upon. *D. sechellia* can utilize fruits other than *M. citrifolia* fruit, but *D. simulans* cannot feed or reproduce upon ripe *M. citrifolia* fruit (R'Kha *et al*. 1991). In our first pilot mate choice experiments, for instance, we used thawed ripe *M. citrifolia* fruit instead of yeast paste. All the *D. simulans* flies died, a result that is consistent with previous work (R'Kha *et al*. 1991). Reproduction on *M. citrifolia* fruits requires resistance to *M. citrifolia* fruit toxin, and we found that the loci that influence interspecific mating in females are found near major-effect resistance loci in a couple of cases. The locus that contributes to interspecific mating of females, QTL-3R_*sim*_B, is just 70 kilobase pairs from the peak of a dominant locus of major effect (>44%) for larval resistance, and the peak of QTL-3R_*sech*_ is just approximately 1.5 Mbp from a recessive larval resistance locus at 3R: ∼15.1 Mbp (Huang and Erezyilmaz 2015). If hybrid female adults bearing one *D. simulans* allele at QTL-3R_*sech*_ or 2 *D. simulans* alleles at QTL-3R_*sim*_B reproduce on *M. citrifolia*, their offspring are far less likely to survive the *M. citrifolia* fruit toxin since they are unlikely to bear the neighboring *D. sechellia* resistance loci. Theory predicts (Servedio and Bürger 2020; Servedio and Hermisson 2020), and genetic studies find that for species that have diverged with gene flow, loci that confer reproductive success are near loci that confer adaptation to the local environment (Hawthorne and Via 2001; Merrill *et al*. 2011; Bay *et al*. 2017). In a landmark study of incipient species of pea aphid that were adapted to 2 different host plants, linkage between mate choice (measured as host plant acceptance) and adaptation (measured as fecundity) was mapped to 4 clusters housed on 2 chromosomes (Hawthorne and Via 2001). The degree of linkage found in the pea aphid study of Hawthorne and Via (2001) is more extensive than the 2 instances of linkage between the major QTL on 3R with the *M. citrifolia* fruit toxin resistance loci of larvae. However, resistance to *M. citrifolia* fruit toxin is critical for Drosophila to be viable on *M. citrifolia* fruit.

The genetic architecture of interspecific mating in females described here may help to explain how the 2 species diverged, but it will also help to predict the outcome of ongoing hybridization in the Seychelles, where interbreeding between endemic *D. sechellia* and invasive *D. simulans* is underway (Matute and Ayroles 2014). Whether the 2 species become more isolated, blend together, or persist as hybrid swarms will be shaped by the shared influence of extrinsic factors like host fruits, and intrinsic factors like the genetic architecture of interspecific mating. We discovered that some genotypes of hybrid females promote assortative mating in the invasive species and other genotypes that facilitate hybridization with the invasive species. Future field work that samples wild Drosophila populations in the Seychelles will help to follow the fate of this ongoing hybridization.

## Supplementary Material

jkae279_Supplementary_Data

## Data Availability

All strains of Drosophila are available upon request, except for *D. sechellia^D1A1C^*, which has been lost. Raw sequence reads for the 2 reference genomes and the separate genotyped library of hybrids will be deposited before publication on NCBI's SRA, indexed with BioProject number PRJNA874916. The genotype × phenotype files are available at the UK's Environmental Information Data Centre: https://doi.org/10.5285/8dde3529-1cf7-4b0f-907d-a1631f38afd7. The raw data for mating rates are provided at: https://doi.org/10.5285/361621ad-6487-47a4-bf8b-00f78705e593. The *D. simulans* genome is found at: https://www.ebi.ac.uk/ena/browser/view/GCA_000754195.3. The MSG software can be found here: https://github.com/JaneliaSciComp/msg. pull_thin script can be found here: https://github.com/dstern/pull_thin. R/qtl package: https://rqtl.org/. R 3.1.3: https://cran-archive.r-project.org/bin/windows/base/old/3.1.3/. Supplemental material available at G3 online.
