# Supplementary material for: Two major-effect loci influence interspecific mating in females of the sibling species, Drosophila simulans and D. sechellia
Source: G3 (Bethesda). 2024 Nov 28;15(2):jkae279. doi: 10.1093/g3journal/jkae279 (PMC11797031; doi:10.1093/g3journal/jkae279)
Supplement: jkae279_Supplementary_Data [file jkae279_supplementary_data.zip › Fig.S1_G3-2024-405418.pdf]

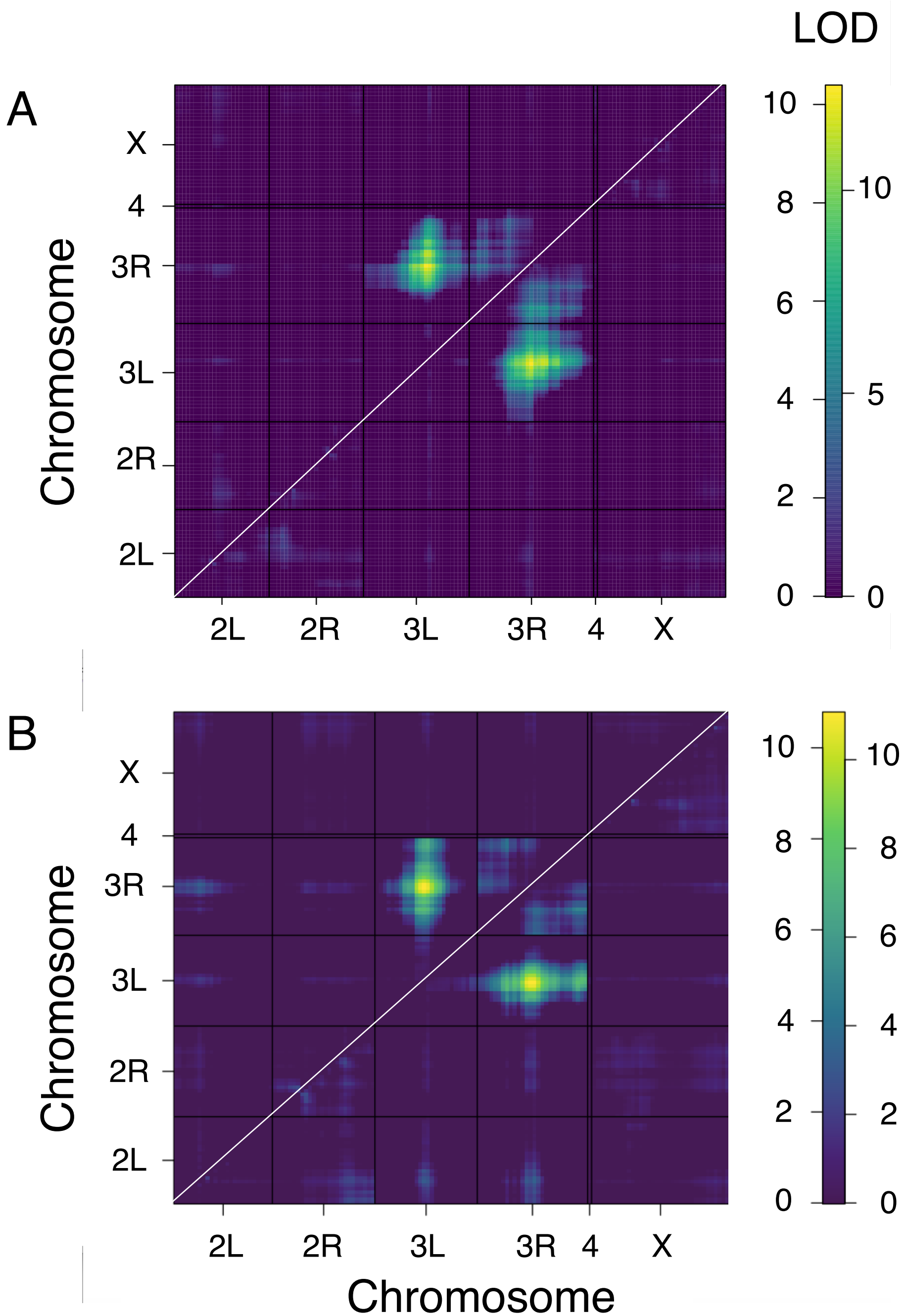

Figure S1. Heat map of LOD scores of two-QTL scans of both backcrosses. A. Two-QTL scan of the *D. simulans* backcross. LOD scores for the improvement of the additive model over a single QTL model ($LOD_{add\text{-}v\text{-}1}$) are graphed in the upper left triangle, and LOD scores for $LOD_{full\text{-}v\text{-}1}$, the improvement of the full (additive + interactive) 2-QTL model over a single-QTL model are given in the lower right triangle B. Two-QTL scan of the *D. sechellia* backcross with ($LOD_{add\text{-}v\text{-}1}$) model in the upper left triangle, and $LOD_{full\text{-}v\text{-}1}$ in the lower right triangle.
