# Supplementary material for: Two major-effect loci influence interspecific mating in females of the sibling species, Drosophila simulans and D. sechellia
Source: G3 (Bethesda). 2024 Nov 28;15(2):jkae279. doi: 10.1093/g3journal/jkae279 (PMC11797031; doi:10.1093/g3journal/jkae279)
Supplement: jkae279_Supplementary_Data [file jkae279_supplementary_data.zip › Fig._S2_G3-2024-405418.pdf]

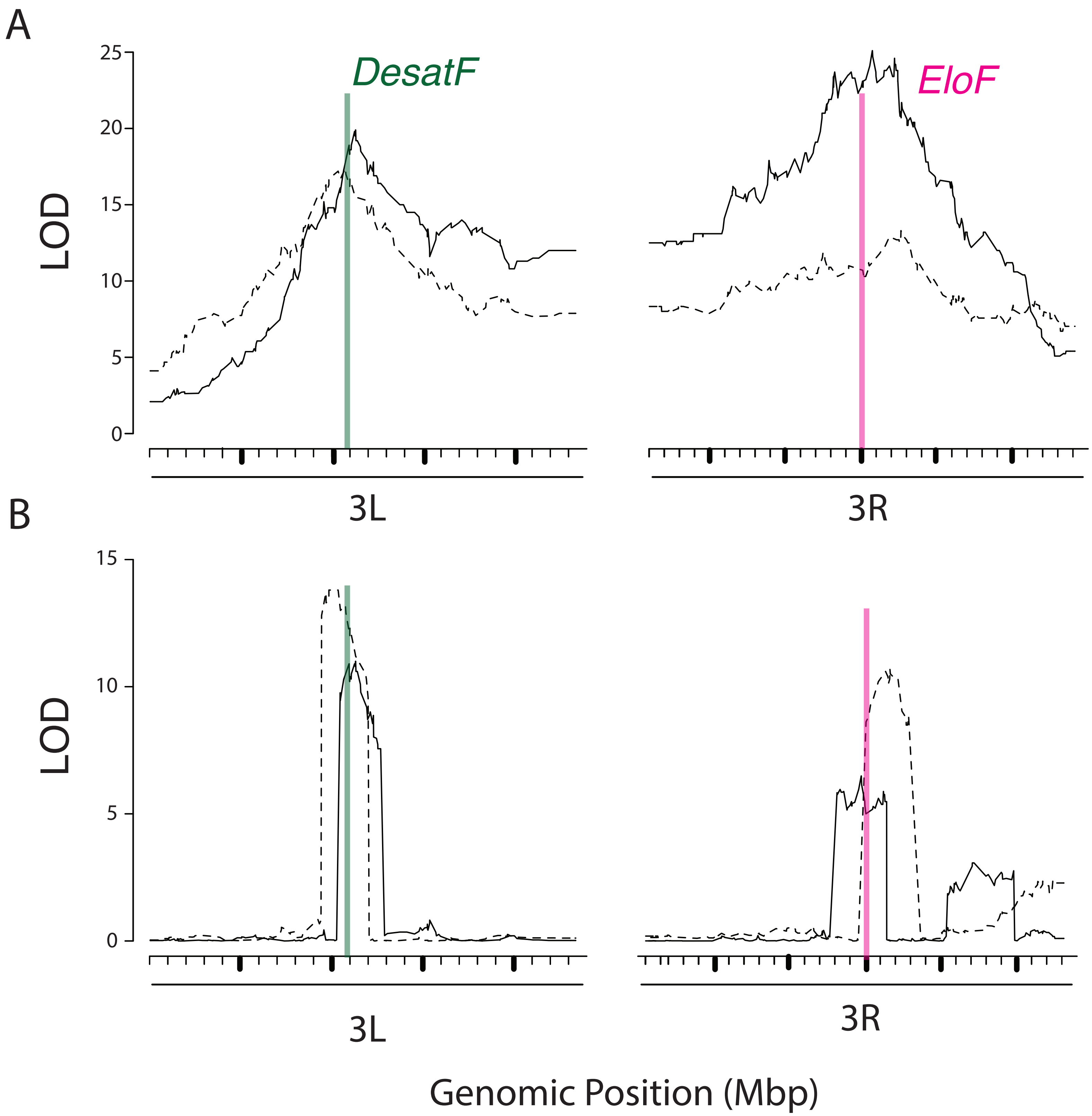

Supplementary Figure 2. Overlay of LOD scores x physical distance (Mbp) of the third chromosome using data produced by A. Interval Mapping and B. Composite Interval. Mapping of the *D. simulans* backcross (solid lines) and the *D. sechellia* backcross (dashed lines). The $p = 0.05$ significance threshold is LOD 2.52 for the *D. simulans* backcross, and $p = 2.57$ for the *D. sechellia* backcross. The locations of *DesaturaseF(DesatF)* and *ElongaseF (EloF*) are marked in green and pink, respectively.
