# Supplementary material for: Two major-effect loci influence interspecific mating in females of the sibling species, Drosophila simulans and D. sechellia
Source: G3 (Bethesda). 2024 Nov 28;15(2):jkae279. doi: 10.1093/g3journal/jkae279 (PMC11797031; doi:10.1093/g3journal/jkae279)
Supplement: jkae279_Supplementary_Data [file jkae279_supplementary_data.zip › File_S8_G3-2024-405418.pdf]

Protocol for Genotyping with desatF/eloF primers

Reaction:

1-5 µL gDNA template(~200ng)

2.5 µL 10 10µM forward primer

2.5 µL 10 10µM reverse primer

25 µL MyTaq Red 2X Mix (Meridian Bioscience)

X µL ddH_2O to 50 µL final volume

In thermocycler:

95C 1 min

Step	Temperature	Time	Cycles
Initial denaturation	95C	1 min	1
Denaturation	95C	15s	30 cycles
Annealing	Tm*	15s	
Extension	72C	10s	

**desatF_for/desatF_rev*: Tm = 60C

* *eloF_for/eloF_rev*: Tm = 62C

PCR reactions were loaded onto a 2% gel and run until separation in size was apparent for control PCR reactions conducted with gDNA from *D. simulans*, *D. sechellia* and F1 hybrid DNA.
