# Supplementary material for: Two major-effect loci influence interspecific mating in females of the sibling species, Drosophila simulans and D. sechellia
Source: G3 (Bethesda). 2024 Nov 28;15(2):jkae279. doi: 10.1093/g3journal/jkae279 (PMC11797031; doi:10.1093/g3journal/jkae279)
Supplement: jkae279_Supplementary_Data [file jkae279_supplementary_data.zip › Supplemental_Legends_G3-2024-405418.docx]

**Supplementary Online Material**

**Fig. S1.** Two-QTL scans of both backcrosses.

**Fig. S2.** Overlay of LOD scores x physical distance (Mbp) of the third chromosome.

**Table S1.** Comparison of QTL models of the *D. simulans* backcross.

**Table S2.** Comparison of QTL models of the *D. sechellia* backcross.

**File S1.** Sequence of 384 bar-coded adapters.

**File S2.** Molecular protocol for production of MSG libraries.

**File S3**. List of genes and D. simulans genome locations within the confidence interval of QTL-3L*_sim_*

**File S4**. List of genes and *D. simulans* genome locations within the confidence interval of QTL-3R*_sim_*A

**File S5.** List of genes and *D. simulans* genome locations within the confidence interval of QTL-3R*_sim_*B

**File S6.** List of genes and *D. simulans* genome locations within the confidence interval of QTL-3L*_sech_*.

**File S7**. List of genes and *D. simulans* genome locations within the confidence interval of QTL-3R*_sech_*.

**File S8**. PCR Genotyping Protocol that was used for females tested in the one-choice tests.
