# Supplementary material for: Two major-effect loci influence interspecific mating in females of the sibling species, Drosophila simulans and D. sechellia
Source: G3 (Bethesda). 2024 Nov 28;15(2):jkae279. doi: 10.1093/g3journal/jkae279 (PMC11797031; doi:10.1093/g3journal/jkae279)
Supplement: jkae279_Supplementary_Data [file jkae279_supplementary_data.zip › Table_S1_G3-2024-405418.pdf]

Table S1. ***QTL locations and models for QTL analyses of the D. simulans backcross.***

Cross	# QTL	% variance (model)¶	LOD of Model*	QTL Location (Mbp)	LOD Drop One†	*p*-value (chi²)	% variance
bc-sim	2	17.86	33.41	~3L:11.31	9.38	$4.91X10^{-11}$	4.67
				~ 3R:14.76	14.28	$5.55X10^{-16}$	7.21
bc-sim	2 + inter-action	18.42	34.57	~3L:11.31	10.55	$2.84X10^{-11}$	5.23
				~ 3R:14.76	15.45	$3.33X10^{-16}$	7.77
				~3L:11.31X~ 3R:14.76	1.16	0.021	0.56
bc-sim	3	19.31	36.43	~3L:11.31	10.47	$3.80 X 10^{-12}$	5.13
				~ 3R:14.76	5.90	$1.89 X 10^{-7}$	2.85
				~ 3R:22.15	3.02	0.00019	1.45
bc-sim	3 + inter-action‡	20.04	38.01	~3L:11.31	12.06	$8.77x10^{-13}$	5.88
				~ 3R:14.76	6.11	$1.13x10^{-7}$	2.93
				~ 3R:22.15	4.61	$2.48x10^{-5}$	2.20
				~ 3L:11.31 X ~ 3R:22.15	1.59	0.0069	1.59
bc-sim	3 + inter-action	19.41	36.64	~ 3L:10.95	6.54	$4.08X10^{-8}$	3.16
				~ 3R:16.23	13,41	$3.92X10^{-14}$	6.62
				~ 3R:7.87	1.49	0.00079	1.49
				~ 3R:16.23 X ~ 3R:7.87	1.33	0.00035	1.33
bc-sim	3 + inter-action	18.97	35.71	~ 3L:10.95	6.16	$9.9 X10^{-8}$	3.0
				~ 3R:16.23	13.72	$1.91 X 10^{-14}$	6.82
				~ 3R:5.54	2.18	0.0066	1.05
				~ 3R:16.23 X ~ 3R:5.54	1.09	0.0031	0.91
bc-sim	5 + 2 inter-action	19.46	36.75	~ 3L:10.95	6.06	$1.26 X 10^{-7}$	2.93
				~ 3R:16.23	13.50	$2.0 X 10^{-13}$	6.67
				~ 3R:7.87	1.03	0.094	0.49
				~ 3R:5.54	0.10	0.79	0.50
				~ 3R:16.23 X ~ 3R:7.87	0.92	0.04	0.44
				~ 3R:16.23 X ~ 3R:5.54	0.77	0.55	0.37

¶Estimated proportion of the phenotype variance explained by all the terms in the model
*Relative to the null model, with no QTL
†Log-likelihood ratios comparing the full model to a model with the specified QTL removed.
‡ The favored model. Inclusion of additional QTL or interactions reduced % variance of QTL in the model and did not significantly improve the score of our model.
