# Supplementary material for: Two major-effect loci influence interspecific mating in females of the sibling species, Drosophila simulans and D. sechellia
Source: G3 (Bethesda). 2024 Nov 28;15(2):jkae279. doi: 10.1093/g3journal/jkae279 (PMC11797031; doi:10.1093/g3journal/jkae279)
Supplement: jkae279_Supplementary_Data [file jkae279_supplementary_data.zip › Table_S2_G3-2024-405418.pdf]

Table S2. QTL locations and models for QTL analyses of the D. sechellia backcross

Cross	# QTL	% variance (model)¶	LOD of Model*	QTL Location (Mbp)	LOD Drop One†	*p*-value (Chi^2)	% variance
bc-sech	2[‡]	17.30	28.47	3L:~10.48	14.67	2.22×10^{-16}	8.51
				3R:~16.63	10.43	4.23×10^{-12}	5.96
bc-sech	2	17.56	28.93	3L:~10.35	15.13	2.0×10^{-16}	8.76
				3R:~16.63	11.13	8.08×10^{-13}	6.36
bc-sech	3[§]	17.99	29.72	3L:~10.35	13.55	2.89×10^{-15}	7.84
				3R:~16.63	4.06	1.54×10^{-5}	2.30
				3R:~11.49	0.79	0.06	0.43
bc-sech	3	18.80	31.19	3L:~10.35	15.05	2.0×10^{-16}	8.58
				3R:~16.63	6.29	7.31×10^{-8}	3.48
				3R:~26.41	2.27	0.001	1.24
bc-sech	4	19.55	32.60	3L:~10.35	13.29	5.22×10^{-15}	7.46
				3R:~16.63	1.34	0.013	0.72
				3R:~11.49	1.40	0.011	0.76
				3R:~26.41	2.88	0.00027	1.56
bc-sech	4 + inter-action **	20.38	34.14	3L:~10.35	13.61	2.44×10^{-15}	7.57
				3R:~16.63	1.66	0.0057	0.89
				3R:~11.49	2.95	0.0011	1.58
				3R:~26.41	4.42	0.0013	2.39
				3R:~11.49 X 3R:~26.41	1.55	0.03	0.83

¶Estimated proportion of the phenotype variance explained by all the terms in the model
*Relative to the null model, with no QTL
†Log-likelihood ratios comparing the full model to a model with the specified QTL removed.
‡ We first asked which pair of QTL with nearly identical location performed best (the 3L/3R pair that was suggested by the one QTL scan or those significant loci produced by two-QTL scans.
§ We then asked which of the additional locations on 3R could improve model fit.
**The favored model. Inclusion of additional QTL or interactions reduced % variance of individual QTL in the model but only slightly improved the score of our model.
