# Supplementary material for: Two major-effect loci influence interspecific mating in females of the sibling species, Drosophila simulans and D. sechellia
Source: G3 (Bethesda). 2024 Nov 28;15(2):jkae279. doi: 10.1093/g3journal/jkae279 (PMC11797031; doi:10.1093/g3journal/jkae279)
Supplement: jkae279_Supplementary_Data [file jkae279_supplementary_data.zip › File_S1_G3-2024-405418.doc]

**Illumina Deep Sequencing Mapping Protocol**

**Dilute DNA to a standard concentration.**

Generate an excel file with listing the volume of water required to dilute each sample to a DNA concentration of 2 ng/ul. (this is flexible, you need to deposit 5-10 ng of DNA per sample for the digestion, so calculate a workable concentration). Create a DNA dilution plate.

**Digest the genomic DNA samples with Mse I.**

Prepare a digestion master mix. For 20 ul reaction, add per sample:

2ul	NEB Buffer 4

0.2 ul	BSA (100X)

0.3 ul	MseI (3.3 U)

10.5 ul	water

13 ul	Total

+ 7 ul sample (around 10 ng DNA)

Incubate at 37C for 3 hours in a thermocycler. Inactivate at 65C for 20 minutes in a thermocycler.

**Ligate bar-coded adapters and pool samples.**

Add to each 20 ul sample of digested genomic DNA:

5 ul	ligase Buffer (10X)

0.2 ul	T4 ligase (80 U)	400,000 U/ml (keep on ice)

23.8 ul	water

29 ul	Total

1 ul	adapter oligos (5 uM stock solutions)

1. Add 29 ul solution into each 96-well.

2. Spin down the adapter oligos plate and add 1 ul of unique adapter oligos for one plate. Keep track of the color of adapter oligos.

Total volume 50 ul.

Ligate in a thermocycler at 16C for 3 hours. Disable heating the lid.

**Ethanol precipitate.**

1. Prepare a 50 ml falcon polypropylene tube.
2. Add 100 ul (2 volumes) of Ethanol into each sample in the PCR plate (reaction stops immediately after mixing with ethanol).
3. Transfer the total volume of 150 ul (each well) into a reservoir.
4. Transfer all liquid in the reservoir into a 50 ml tube. Label it clearly.
5. Add 500 ul of 3M sodium acetate (NaOAc pH 5.2) into the 50 ml tube.
6. Chill overnight at -20C in the freezer.
7. Pellet the precipitate by centrifuging at 4000X for 30 min.
8. **Carefully** pour off the supernatant, do not disturb the pellet.
9. Wash the sides of the tube with 1 volume cold 70% ethanol (15ml for one tube) and dump the ethanol **carefully.**
10. Pour 12 ml of 70% ethanol into the tube carefully (don’t touch the rim of DNA tube)
11. Centrifuge at 4000X for another 5 minutes and remove the supernatant (use a pipette to remove the last residuals but don’t touch the pellet).
12. Air dry (to get rid of the ethanol completely).
13. Re-suspend the pellet in 200 ul TE (Tris EDTA pH8) (Do not draw the pellet into the barrel of tips and make sure all of the DNA go into solution, it is OK to have two tubes with 200 ul DNA in each one).

**Phenol: Chloroform Extraction**

- To stop the reaction, add one volume (200 ul for 200 ul DNA, 400 ul for 400 ul DNA) of phenol:chloroform:IAA. (At 4 C, the phenol : chloroform solution has two layers – be sure to take bottom layer.)
- Mix voraciously; spin for 5 minutes at maximum speed. Do not let it sit for over 1 minute after spinning.
- Remove aqueous layer (top layer) to a new tube. (Do not touch the interface and always leave some aqueous layer behind).
- Dump the bottom layer into phenol:chloroform liquid waste container.
- Add equal volume of chloroform: IAA (200 ul for 200 ul DNA).
- Mix; spin for 5 minutes at maximum speed.
- Remove aqueous (top) layer to a new tube. Dump bottom layer into waste container. Label the new tube with DNA.

Usually you have 160 ul DNA out of the beginning 200 ul after the Phenol: Chloroform Extraction.

**Bead purify using the Agencourt AMPure PCR purification kit.**

- Swirl bottle to resuspend beads by vortex, make sure no sediment on the bottom (it is a small bottle in the fridge not a big one).
- Add1.5 volume (300 ul for 200 ul DNA) of beads, mix it by pipetting 15 times. (Do not vortex).
- Incubate at Room Temperature 5 mins.
- Place two tubes at two diagonal ends **on magnet** 10 mins.
- Aspirate and discard supernatant.
- Add 2 volume (400 ul for 200 ul DNA) of 70% EtOH.
- Incubate at RT 1 min.
- Aspirate and discard supernatant.
- Repeat EtOH wash.
- Air dry for at **MOST** 5 mins (Over dry is dangerous).
- **Off the magnet**, re-suspend in 0.5 volume (100 ul for 200 ul DNA) of TE (Tris-EDTA).
- Incubate at RT 1 min.
- Place **back on magnet** for 5 mins.
- Transfer supernatant to a new 1.5 ml tube. Label it with the name of this step.
- Put it in the freezer for storage.

**Run pooled sample on a gel.**

Agarose 1.8%/0.2% GTG in TAE 1X.

For 60 ml TAE 1X, we use 1.08 g of Agarose and 0.12 g of GTG.

Run up your DNA with 50 bp ladder. (Fermentas Generuler 50 bp). Use 1X TAE for running buffer. For each 100 ul DNA, add 20 ul of 6X Dye (total 120 ul), load 60 ul of sample into each well. Load 10 ul of 50 bp ladder.

Electrophoresis at 70 V for 1-2 hours.

Cut band between 250-300 bp ladder bands on the transilluminator (keep 200-250 bp or 300-400 bp just in case).

Put each piece of Gel into each1.5 ml tube.

Use QIAquick Gel Extraction Kit to recover DNA.

**QIAquick Gel Extration Kit**

Notes before starting

- The yellow color of Buffer QG indicates a pH < 7.5.
- Add ethanol (96–100%) to Buffer PE before use (see bottle label for volume).
- Isopropanol (100%) and a heating block or water bath at 50°C are required.
- All centrifugation steps are carried out at 13,000 rpm (~17,900 x g) in a
- conventional table-top microcentrifuge.

1. Excise the DNA fragment from the agarose gel with a clean, sharp scalpel.

Minimize the size of the gel slice by removing extra agarose.

2. Weigh the gel slice in a colorless tube. Add 3 volumes of Buffer QG to 1 volume of

gel (100 mg gel ~ 100 μl).

For example, add 300 μl of Buffer QG to each 100 mg of gel. The maximum amount of gel slice per QIAquick column is 400 mg; for gel slices >400 mg use more than one QIAquick column. For >2% agarose gels, add 6 volumes of Buffer QG.

3. Incubate at 50°C for 10 min (or until the gel slice has completely dissolved). To help

dissolve gel, mix by vortexing the tube every 2–3 min during the incubation.

IMPORTANT: Solubilize agarose completely. For >2% gels, increase incubation time.

4. After the gel slice has dissolved completely, check that the color of the mixture is

yellow (similar to Buffer QG without dissolved agarose).

If the color of the mixture is orange or violet, add 10 μl of 3 M sodium acetate,

pH 5.0, and mix. The color of the mixture turns yellow.

5. Add 1 gel volume of isopropanol to the sample and mix.

For example, if the agarose gel slice is 100 mg, add 100 μl isopropanol.

6. Place a QIAquick spin column in a provided 2 ml collection tube. To bind DNA, apply the sample to the QIAquick column, and centrifuge for 1 min.

The maximum volume of the column reservoir is 800 μl. For sample volumes of more

than 800 μl, simply load and spin again.

8. Discard flow-through and place QIAquick column back in the same collection tube.

Collection tubes are re-used to reduce plastic waste.

9. To wash, add 750 ul of Buffer PE to QIAquick column and centrifuge for 1 min.

10. Discard the flow-through and place the QIAquick column back into the same tube. Centrifuge the QIAquick column in the provided 2 ml collection tube for an additional 1 min to remove residual wash buffer.

12. Place QIAquick column into a clean 1.5 ml microcentrifuge tube.

13. To elute DNA, add 50 μl of Buffer EB (10 mM Tris·Cl, pH 8.5) or H2O to the center of the QIAquick membrane and centrifuge the column for 1 min. For increased DNA concentration, add 30 μl buffer EB to the center of the QIAquick membrane, let the column stand for 1 min, and then centrifuge for 1 min. After the addition of Buffer EB to the QIAquick membrane, increasing the incubation time to up to 4 min can increase the yield of purified DNA.

IMPORTANT: Ensure that the elution buffer is dispensed directly onto the QIAquick

membrane for complete elution of bound DNA.

**Amplify bar-coded fragments using the Phusion PCR kit and FC1 and FC2 primers.**

Per 25 ul reaction:

5 ul	5X Phusion. Buffer

0.25 ul	enzyme (Add until the last step)

0.5 ul	10mM dNTP

1.25 ul	10uM FC1

1.25 ul	10uM FC2

5 ul	template DNA

11.75 ul	water

Run the FC_PCR program in the PCR machine.

The program should be:

1. 98 C	30 sec

2. 98 C	10 sec

3. 60 C	15 sec

4. 72 C	15 sec

5. 72 C	7	min

6. 4 C	--	2-4: total x cycles

Run the minimum # of PCR cycles required to generate sufficient DNA for the flow cell.

Try 0 cycle, 5 cycles, 10 cycles, 15 cycles, 20 cycles.

Run the DNA on the Gel, with1.8%Agarose/0.2% GTG in 60 ml TAE 1X. We use 1.08 gram agarose and 0.12 gram of GTG.
